# Targeting *Helicobacter pylori* enzymes using *Viscum album* L. extract: *in silico* molecular docking and *in vitro* study

**DOI:** 10.3389/fcimb.2025.1690969

**Published:** 2026-01-12

**Authors:** Ioana Alexandra Cardos, Catalina Danila, Galal Yahya, Noura M. Seleem, Rasha A. Mosbah, Moataz A. Shaldam, Simona Ioana Vicas, Kamel Metwally, Simona Daniela Cavalu

**Affiliations:** 1 Doctoral School of Biomedical Science, University of Oradea, Oradea, Romania; 2 Faculty of Medicine and Pharmacy, University of Oradea, Oradea, Romania; 3 Department of Microbiology and Immunology, Faculty of Pharmacy, Zagazig University, Zagazig, Egypt; 4 Molecular Biology Institute of Barcelona (IBMB), Consejo Superior de Investigaciones Científicas (CSIC), Barcelona, Spain; 5 Infection Control Unit, Zagazig University Hospital, Zagazig, Egypt; 6 Military Medical Academy, Cairo, Egypt; 7 Department of Pharmaceutical Chemistry, Faculty of Pharmacy, Kafrelsheikh University, Kafrelsheikh, Egypt; 8 Faculty of Environmental Protection, University of Oradea, Oradea, Romania; 9 Department of Pharmaceutical Chemistry, Faculty of Pharmacy, University of Tabuk, Tabuk, Saudi Arabia

**Keywords:** *H. pylori*, mistletoe, molecular docking, phenolic compounds, urease inhibitor

## Abstract

*Viscum album* L. (mistletoe) is a hemiparasitic plant known for its wide range of bioactive compounds, including phenolics, flavonoids, and lectins, which contribute to its diverse pharmacological properties. In the present study, we focused on identifying and quantifying the phenolic compounds present in *V. album* L. leaf extracts and evaluating their potential as inhibitors of key *Helicobacter pylori* enzymes through both *in silico* and *in vitro* approaches. Using molecular docking, we assessed the binding affinity and stability of selected mistletoe’s phytochemicals with specific *H. pylori* targets, including peptide deformylase, shikimate pathway enzymes, and urease. Additionally, to complement the computational findings, we conducted an *in vitro* assay to evaluate the anti-urease activity of the crude *V. album* L. extract against the urease activity of *Proteus mirabilis*. The extract demonstrated significant inhibitory activity, indicating its potential as a natural urease inhibitor at a concentration of 0.0125 mg/mL, leading to a marked reduction in urease-mediated crystal formation in artificial urine. Furthermore, the extract exhibited broad-spectrum anti virulence effects by suppressing biofilm formation in *Staphylococcus aureus* and *Escherichia coli*, and inhibiting protease activity in *S. aureus* and *Pseudomonas aeruginosa*. Together, these findings highlight *V. album* phenolics as promising dual-action natural inhibitors that not only target essential metabolic enzymes but also attenuate virulence factors critical for pathogenesis. This integrated strategy positions *V. album* as a strong candidate for the development of plant-based therapeutics against multidrug-resistant pathogens with possible applications in the treatment of *H. pylori*-related gastrointestinal disorders.

## 1 Introduction

*H. pylori* is a Gram-negative, spiral-shaped bacterium responsible for a chronic, transmissible infectious disease that induces progressive gastric inflammation. It is implicated in several gastrointestinal disorders, including gastric and duodenal ulcers, and is a well-established risk factor for gastric cancer (Rawla and Barsouk, 2019; Kim and Chung, 2020). As a result, the International Agency for Research on Cancer (IARC) has classified *H. pylori* as a Group 1 carcinogen, based on comprehensive laboratory and epidemiological evidence (Savoldi et al., 2018). The pathogenicity of *H. pylori* is largely attributed to its virulence factors, particularly the cytotoxin-associated gene A (CagA) protein, which promotes inflammation and has carcinogenic potential, and the vacuolating cytotoxin A (VacA), which invades epithelial cells and induces apoptosis (Hatakeyama and Higashi, 2005; Baltrus et al., 2009). Despite decades of research, a definitive treatment for complete eradication remains elusive, primarily due to increasing antibiotic resistance, often resulting from chromosomally encoded mutations (Boyanova et al., 2023). A key survival strategy of *H. pylori* is its production of urease, which can constitute uto 6% of its total protein content. Urease neutralizes gastric acid by generating ammonia, enabling the bacterium to survive in the acidic environment of the stomach. In addition, *H. pylori* utilize chemotaxis to migrate toward less acidic regions of the gastric mucosa (Boyanova et al., 2023).

Current treatment regimens rely on a limited arsenal of antibiotics, including clarithromycin, amoxicillin, metronidazole, fluoroquinolones (levofloxacin, norfloxacin), tetracycline, and rifabutin. However, standard triple therapy—comprising a proton puminhibitor (PPI), amoxicillin, and clarithromycin—has lost efficacy in many regions due to rising resistance (Kim and Chung, 2020). Bismuth- and non-bismuth-based quadruple therapies offer better eradication rates (>80%) and safety profiles, though they can cause gastrointestinal side effects (Andreev, 2017; Chey et al., 2017; Zagari et al., 2023). Rifabutin-based triple therapy (amoxicillin, rifabutin, and a PPI) has shown superior efficacy but is expensive, poses risks for tuberculosis resistance, and carries a small risk of myelotoxicity (Gisbert and Calvet, 2012; Kim and Chung, 2020).

Fluoroquinolone-based regimens serve as second-line treatments where bismuth is unavailable or clarithromycin resistance is prevalent. However, their efficacy against gyrA-mutation-positive strains remains unclear (Kim and Chung, 2020; Boyanova et al., 2023). Recently, potassium-competitive acid blockers (PCABs), such as vonoprazan, have emerged as superior alternatives to PPIs, offering rapid and sustained acid suppression without requiring activation by gastric acid (Cardos et al., 2021), and avoiding adverse effects associated with prolonged use of PPIs like intestinal dysbiosis and small intestinal bacterial overgrowth (SIBO) (Dominiczak et al., 2024). Vonoprazan-based therapy has demonstrated greater cost-effectiveness and a low incidence of adverse effects (4.4%) compared to conventional first-line treatments (Tanabe et al., 2017; Yunusa and Love, 2023).

Nonetheless, eradication failure is often multifactorial, influenced by sociodemographic and clinical variables, including patient compliance and comorbidities such as diabetes, hypertension, and chronic liver or kidney diseases—though these relationships remain controversial (Gebeyehu et al., 2019). Given the growing threat of antimicrobial resistance and the cost burden of conventional therapies, interest in alternative strategies has increased. Natural products, particularly plant-based remedies with anti-inflammatory, antioxidant, and antimicrobial properties, offer promising therapeutic potential (Safavi et al., 2015; Cardos et al., 2021). Phytochemicals are chemically diverse and are often associated with lower toxicity profiles compared to certain synthetic agents, making them attractive candidates for drug discovery. Numerous plant extracts—including berberine, *Curcuma longa*, ginseng, propolis, *Allium sativum*, and *Prunus dulcis*—have demonstrated *in vitro* and *in vivo* anti-*H. pylori* effects (Jiang et al., 2016; Lawal et al., 2011; Cardos et al., 2021). Compounds such as phenolic acids (cinnamic, caffeic, ferulic, syringic, p-coumaric, protocatechuic, gentisic, gallic), tannins, and sulforaphane have shown promise, though their precise mechanisms remain largely undefined. Notably, one of *H. pylori*’s major survival tools is urease, making its inhibition a viable therapeutic target (Jadhav et al., 2013). Advances in genomics, proteomics, and molecular modelling have enabled the identification of novel drug targets. Molecular docking, in particular, provides valuable insight into ligand–protein interactions, supporting the design of targeted urease inhibitors (Xiao et al., 2012).

*V. album* L. (mistletoe), a hemiparasitic plant, is rich in bioactive compounds and has been used as a complementary treatment for hypertension, atherosclerosis, arthritis, diabetes, and cancer (Kleszken et al., 2022). Its therapeutic effects are attributed to the synergistic interactions of various secondary metabolites, including those with antioxidant properties (Karagöz et al., 2016; Segneanu et al., 2022). Importantly, its phytochemical profile and biological effects vary depending on the host tree (Vica et al., 2011).

In this study, we aimed to screen *V. album* L. leaf extracts from northwestern Romania to identify and quantify phenolic compounds responsible for biological activity. We also investigated their potential anti-*H. pylori* activity using *in silico* approaches employing molecular docking simulations. We evaluated the *in vitro* anti virulence potential of *V. album* extract against clinically relevant pathogens. Specifically, *Proteus mirabilis* was selected for urease inhibition assays because its urease is structurally conserved and functionally analogous to *H. pylori* urease, making it a suitable model for assessing urease-targeting strategies. Biofilm inhibition was evaluated in *S. aureus* and E. coli, two major opportunistic pathogens where biofilm formation contributes to persistence and antibiotic tolerance. Protease inhibition was assessed in *S. aureus* and *P. aeruginosa*, as secreted proteases are key virulence factors implicated in tissue damage, immune evasion, and chronic infection. Including these pathogens enables the assessment of *V. album* extract across multiple virulence mechanisms and provides a broader context for its potential as a natural antivirulence agent. By integrating *in silico* enzyme-targeting analyses with *in vitro* assays of virulence suppression, this study aimed to provide a comprehensive evaluation of *V. album* as a dual-action natural antimicrobial candidate targeting both essential bacterial enzymes and virulence-associated traits.

## 2 Materials and methods

### 2.1 Biological material and sample preparation

Mistletoe leaves were harvested in May 2022 from apple trees (*Malus domestica* Borkh.) located near Sânnicolau de Munte, Bihor County, Romania. The collecting site is situated on County Road DJ 767A, around 44 kilometers from Oradea, at an altitude of 132 m above sea level (coordinates: 47°18′13″ N, 22°08′12″ E). This area belongs to the Western Plain and is defined by a temperate-continental climate with oceanic influences, especially resulting from prevailing westerly winds. A mistletoe (*V. album* L. subs *album*) specimen, registered under the NYBG Steere Herbarium’s PUO 05361 number, was stored in the Herbarium of the Faculty of Medicine and Pharmacy in Oradea, Romania. After collection, the leaves were air-dried in the dark and powdered. The extract was prepared by mixing 0.5 g of dry powder sample with 5 mL of 70% ethanol, vortexed for 1 min, followed by ultrasonic treatment in a Bandelin Sonorex ultrasonic bath (35 kHz, nominal power 60–120 W) for 30 min at approximately 30 °C. The obtained extract was centrifuged at 10,000 rpm for 10 min, and the supernatant, containing extracted polyphenols, was filtered through a nylon filter (pore size 0.45 µm) before being subjected to the HPLC analysis.

### 2.2 Bacterial strain, media, and chemicals

The bacterial strains used in this study were *Proteus mirabilis* (strain HI4320), *S. aureus* ATCC 6538, *E. coli* ATCC 25922, and *Pseudomonas aeruginosa* PAO1. Microbiological media like Tryptone soya broth (TSB) were purchased from Oxoid (Hampshire, UK). Stuart’s Urea Broth for urease assay was prepared according to (Stuart et al., 1945; MacFaddin, 2000), where 0.1 g of yeast extract, 9.1 g of Potassium phosphate, monobasic, 9.5 g of Potassium phosphate, dibasic, 20 g of urea, and 0.01 g of phenol red were dissolved in 1 L of distilled water and filter sterilize (0.22-mm pore size). The prepared medium had a yellow-orange color and was stored in the refrigerator at 4–8 °C until use. The medium was not heated to avoid urea decomposition. All chemicals were of pharmaceutical grade.

### 2.3 Identification and quantification of phenolic compounds from mistletoe leaves samples by HPLC-DAD-MS (ESI+)

Qualitative and quantitative analysis was carried out using HPLC Agilent 1200 equipped with quaternary pomp, degassing system, autosampler and UV-VIS detector (DAD) coupled with MS spectrometer (Agilent model 6110, Agilent Techologies, CA, USA). Kinetex XB C18 column (4.6 mm x 150 mm, particle size 5 μm, Phenomenex, Torrance, CA, USA) was employed, while the phases were set uof eluent A (water + 0.1% acetic acid) and eluent B (acetonitrile + 0.1% acetic acid) using the following gradient schedule (total time 30 min, T= de 25 ˚C, flow 0.5 mL/min): 0 min, 5% B; 0–2 min, 5% B; 2–18 min, 5%-40% B; 18–20 min, 40%-90% B; 20–24 min, 90% B; 24–25 min, 90%-5% B; 25–30 min, 5% B.

Mass spectrometric detection was conducted in scan mode, utilizing the following source parameters: gas temperature of 350 °C, nitrogen gas flow rate of 7 L/min, nebulizer pressure of 35 psi, capillary voltage of 3000 V, and fragmentor voltage of 100 V. The mass range was established at m/z 120–1500. Diode-array detection (DAD) was performed at wavelengths of 280 nm and 340 nm. Data acquisition and processing were conducted with Agilent ChemStation software (version B.02.01 SR2; Agilent Technologies, Santa Clara, CA, USA).

The reagents: acetonitrile, HPLC purity, purchased from Merck (Germany); ultra-pure water purified with Direct-Q UV, Millipore (USA); chlorogenic acid standard (>98% HPLC), gallic acid (>99% HPLC) and rutin (>99% HPLC) were purchased from Sigma (USA).

For the quantitative determinations, a calibration curve was obtained using gallic acid standard ((R^2^ = 0.9978), LOD = 0.35 μg/mL, LOQ = 1.05 μg/mL)), chlorogenic acid standard ((R^2^ = 0.9937), LOD = 0.41 μg/mL, LOQ = 1.64 μg/mL) and rutin standard ((R^2^ = 0.9981), LOD = 0.21 μg/mL, LOQ = 0.84 μg/mL) respectively. Phenolic compounds were identified by comparing their retention times, UV–Vis spectra, and mass fragmentation patterns with standards and reference data reported in the scientific literature. Detection at 280 nm was employed for phenolic acids, flavanol monomers, and proanthocyanidin polymers, based on their spectral features, whereas detection at 340 nm was used for hydroxycinnamic acids and flavonols. The HPLC chromatograms, recorded at 280 nm and 340 nm and the calibration curves are presented in the **Supplementary Material S1**.

### 2.4 Molecular docking and computational analysis

The crystal structures of target proteins of *H. pylori* were obtained from the protein data bank (PDB). The docking study was carried out on caffeic acid, gallic acid, naringenin, quercetin, apigenin, and selenium azelate (1:1) azelate using AutoDock Vina (Trott and Olson, 2010). Ligand structures were drawn into Marvin Sketch V22.2 (Chemaxon docs, 2025), and the most energetically favored conformer was exported as a (*.pdb) file format. Molecules of water were removed, adding hydrogen assign Gasteiger charges were performed using AutoDock tools. The centers and sizes of the grid boxes used to define the active site for each receptor are shown in **Table 1**. Docking was performed with an exhaustiveness level of 18, ensuring adequate conformational sampling of ligand orientations. For each ligand, 10 docking poses were generated, and the pose exhibiting the lowest binding free energy (highest docking score) was selected for subsequent analysis of molecular interactions. The 3D visualization and 2D schematic presentation were generated by BIOVIA Discovery Studio Visualizer (Souza et al., 2024).

**Table 1:** Target proteins in *H. pylori*, their PDB code and grid box information.

Protein	Urease	Chorismate synthase	Peptide deformylase	Shikimate kinase
PDB Code [Ref]	1E9Y (Sketch, 2024)	1UMF (BIOVIA, 2024)	2EW5 (Hassan et al., 2025)	3N2E (Rahman et al., 2024)
Grid coordinates (x, y, z)	128.0, 129.1, 86.8	37.3, 45.6, 31.1	-79.0, -64.9, 49.8	38.2, 17.8, 13.8
Grid Size (x, y, z)	11.7, 13.1, 12.0	14.5, 15.2, 17.9	19.2, 15.4, 23.7	23.2, 14.3, 21.0

### 2.5 Minimum inhibitory concentration determination

The MIC of *V. album* extract against *Proteus mirabilis* (strain HI4320), *S. aureus* ATCC 6538, *E. coli* ATCC 25922, and *Pseudomonas aeruginosa* PAO1 was determined by the broth micro-dilution method (Rahman et al., 2024; Hassan et al., 2025). An overnight bacterial culture of the indicated bacterial strains in TS broth was diluted using sterile phosphate buffer saline (PBS) to 0.5 McFarland standards equivalent turbidity. Then 1:100 dilution (in sterile TSB) of the bacterial suspension was prepared. Serial dilutions of *V. album* (10 mg/mL –1.24 µg/mL) in sterile TSB were prepared in sterile 96 wells microplates and 50 µL of freshly prepared bacterial suspension was introduced into each well. After incubation for 18 h at 37°C, the results were recorded. The MIC was calculated as the lowest concentration of *V. album* that inhibited the visible growth of *P. mirabilis*.

### 2.6 Urease test

To evaluate the urease inhibitory activity of *V. album* leaf extract against *P. mirabilis*, a broth-based microdilution assay was performed using 96-well microtiter plates according to (Han et al., 2004). A heavy inoculum was prepared from an 18–24-hour pure culture of *P. mirabilis* and standardized in urea broth medium (0.5 McFarland standard, corresponding to approximately 1 × 10^8^ CFU/mL, using sterile urea broth as the diluent). Two-fold serial dilutions of *V. album* extract were prepared across the wells of the plate to obtain a range of concentrations (10 mg/mL –1.24 µg/mL). Each well received the bacterial suspension and was gently mixed to ensure uniformity. Control wells without the extract (positive control) and wells without bacteria (negative control) were included for comparison. The plates were incubated at 35°C under aerobic conditions. Urease activity was assessed by monitoring the color change of the urea broth at 12 hours. A bright pink (fuchsia) color in the well indicated active urease-mediated hydrolysis of urea, producing ammonia and increasing the pH. The extent of color change was visually compared across different extract concentrations. *P. mirabilis*, as a rapid urease-positive organism, produced a strong positive reaction typically within 8 hours and always by 48 hours in the control wells. Wells showing delayed or absent color change in the presence of *V. album* extract were indicative of urease inhibition. The minimum inhibitory concentration (MIC) was determined as the lowest concentration of the extract at which no visible color change occurred.

### 2.7 Artificial urine

Artificial urine was prepared based on the formulation by Griffith et al. (1976) (Griffith et al., 1976), widely accepted in previous studies (Torzewska et al., 2003; Prywer et al., 2018) with slight modifications. The composition of the artificial urine is summarized in **Table 2**. All chemicals are of reagent-grade (Sigma Aldrich, Darmstadt, Germany). The solution was freshly prepared prior to each experiment and its pH was adjusted to the desired value using 1 M hydrochloric acid (HCl) and 1 M sodium hydroxide (NaOH), adjusted to a pH of 5.8, and sterilized using a 0.2 μm pore-size membrane filter (Fisherbrand, Fischer Scientific ™, Spain). Its composition reflects the average concentrations found in normal human urine over a 24-hour period.

**Table 2:** Composition of artificial urine.

Component	Chemical formula	Concentration (g/L)
Calcium chloride dihydrate	CaCl_2_·2H_2_O	0.651
Magnesium chloride hexahydrate	MgCl_2_·6H_2_O	0.651
Sodium chloride	NaCl	4.6
Sodium sulfate	Na_2_SO_4_	2.3
Sodium citrate	Na_3_C_6_H_5_O_7_	0.65
Sodium oxalate	Na_2_C_2_O_4_	0.02
Potassium dihydrogen phosphate	KH_2_PO_4_	2.8
Potassium chloride	KCl	1.6
Ammonium chloride	NH_4_Cl	1.0
Urea	CH_4_N_2_O	25.0
Creatinine	C_4_H_7_N_3_O	1.1
Tryptic soy broth	—	10.0

### 2.8 Crystal growth experiments

To investigate the impact of *V. album* extract on crystallization of struvite and apatite, in a sterile 96 well polystyrene microplate with a clear flat bottom (Greiner Bio One, Madrid, Spain), 150 µL of synthetic urine was inoculated with *Proteus mirabilis* (strain HI4320) at a final concentration of 10^5^ CFU/mL. The tested extract was added at concentrations of 0.08, 0.04, and 0.02 mg/mL. A control sample, consisting of bacteria suspended in synthetic urine without any additives, was also prepared. All samples were incubated at 37 °C for 24 hours. Crystals were observed using AF7000 Motorized Widefield Microscope (LEICA MICROSYSTEMS).

### 2.9 Anti-biofilm

Overnight cultures of bacteria (*S. aureus* ATCC 6538 and *E. coli* ATCC 25922) were grown in tryptone soya broth (TSB) and subsequently diluted with fresh TSB to an optical density (OD_620_) of 0.2. A total of 150 µL of this suspension was transferred into each well of a 96-well microtiter plate. Each test well received a ¼ minimum inhibitory concentration (MIC) dose of the respective drug, dissolved in DMSO. Two control wells were included: one negative control (bacteria-free) and one positive control (containing bacteria with the vehicle only). The plates were incubated at 37 °C for 24 hours. After incubation, planktonic cells were carefully removed, and wells were gently rinsed with deionized water before being air-dried. Adherent bacterial cells were fixed with methanol for 25 minutes and then stained with 1% (w/v) crystal violet for 20 minutes. Excess stain was rinsed off, and the plates were dried. The stained biofilms were visualized using a fully motorized Leica AF7000 microscope equipped with a 20× objective lens.

### 2.10 Anti-protease

To evaluate the inhibitory effects of *V. album* extract on bacterial protease activity, the skim milk agar assay was performed as previously described (Stuart et al., 1945; MacFaddin, 2000). Overnight bacterial cultures (*S. aureus* ATCC 6538 and *P. aeruginosa* PAO1) grown in TSB and treated with each drug at ¼ MIC in a 96-well plate. The cultures were centrifuged at 10,000 × rpm for 20 minutes to pellet the planktonic cells. Supernatants (100 µL) from each well were then transferred into wells punched into 5% skim milk agar plates. The plates were incubated overnight at 37 °C. Protease activity was indicated by clear zones surrounding the wells. Supernatants from untreated bacterial cultures and 1% SDS served as positive controls, while bacteria-free media served as the negative control.

## 3 Results and discussion

### 3.1 Identification and quantification of phenolic compounds from mistletoe leaves samples by HPLC-DAD-MS (ESI+)

The polyphenolic profile of *V. album* L. leaves extract is presented in **Table 3**.

**Table 3:** Identification and quantification of phenolic compounds in *V. album* L. leaves extract (HPLC-DAD-MS (ESI^+^).

Peak no.	R_t_ (min)	UV λ_max_ (nm)	[M+H] ^+^ (m/z)	Compound	Subclass	mg/g dw
1	3.03	265	155	Dihydroxybenzoic acid	Hydroxybenzoic acid	2.107 ± 0.19
2	10.37	323	355,163	3-Caffeoylquinic acid (Neochlorogenic acid)	Hydroxycinnamic acid	0.775 ± 0.09
3	12.02	323	355,163	4-Caffeoylquinic acid (Criptochlorogenic acid)	Hydroxycinnamic acid	0.313 ± 0.02
4	12.32	323	355,163	5-Caffeoylquinic acid (Chlorogenic acid)	Hydroxycinnamic acid	1.413 ± 0.11
5	13.20	330	387,223	Sinapic acid-glucoside	Hydroxycinnamic acid	0.490 ± 0.04
6	13.79	330	475,163	Dicaffeoyl tartaric acid	Hydroxycinnamic acid	0.585 ± 0.05
7	14.32	330	399,223	3-Sinapoylquinic acid	Hydroxycinnamic acid	0.536 ± 0.04
8	14.84	330	399,223	5-Sinapoylquinic acid	Hydroxycinnamic acid	1.044 ± 0.09
9	15.85	255, 360	611,303	Quercetin-rutinoside (Rutin)	Flavonol glycoside	0.171 ± 0.01
10	16.08	255, 360	465,303	Quercetin-glucoside	Flavonol glycoside	0.323 ± 0.02
11	16.83	330	225	Sinapic acid	Hydroxycinnamic acid	0.570 ± 0.05
12	17.27	255, 360	609,303	Quercetin-O-[hydroxymethylglutaryl] hexoside (Quercetin derivative)	Flavonol	0.392 ± 0.03
13	17.39	240, 350	479,317	Isorhamnetin-glucoside	Flavonol	0.270 ± 0.01
14	18.45	240, 350	623,317	Isorhamnetin -O-[hydroxymethylglutaryl] hexoside (Isorhamnetin derivative)	Flavonol	0.458 ± 0.04
15	18.84	240, 350	493,317	Isorhamnetin-glucuronide	Flavonol	0.445 ± 0.03
16	19.39	240, 350	755,317	Isorhamnetin-(dirhamnosyl)-rhamnoside	Flavonol	0.354 ± 0.03
17	19.77	240, 350	625,317	Isorhamnetin-glucosyl-rhamnoside	Flavonol	0.249 ± 0.01
18	20.25	245, 350	493,331	Rhamnazin-glucoside	Flavonol	0.478 ± 0.04
19	20.64	245, 350	639,331	Rhamnazin-rutinoside	Flavonol	0.279 ± 0.01
20	21.39	255, 360	303	Quercetin	Flavonol	0.137 ± 0.01
21	24.31	240, 350	317	Isorhamnetin	Flavonol	0.042 ± 0.003

Among the hydroxycinnamic acids identified in the analyzed sample, including neochlorogenic acid, cryptochlorogenic acid, chlorogenic acid (compounds 2, 3 and 4) and dicaffeoyl tartaric acid (compound 6), all share a common structural motif derived from caffeic acid. Dicaffeoyl tartaric acid has two parts of caffeic acid attached to tartaric acid, and other compounds like chlorogenic acid also have caffeic acid connected to quinic acid. Although derivatives like sinapic acid and its conjugates do not contain caffeic acid directly, they are biosynthetically derived from it through the phenylpropanoid pathway. Since caffeic acid is a key part of many of these compounds and has known biological effects, it was chosen as the main molecule for the molecular docking analysis. Among the flavonols identified in the mistletoe extract, quercetin (compound 20) and its derivatives represent a core molecular structure. The presence of multiple quercetin glycosides (such as quercetin-rutinoside (compound 9) and quercetin-glucoside, compound 10) and quercetin derivatives (e.g., quercetin-O-[hydroxymethylglutaryl] hexoside, compound 12) indicates that quercetin-based structures dominate the flavonol profile of the mistletoe. Furthermore, structurally related methylated derivatives such as isorhamnetin (compound 21) and rhamnazin-glucoside (compound 18) or rhamnazin-rutinoside (compound 19) differ from quercetin mainly by methylation patterns on the flavonoid backbone, preserving the core flavonol skeleton. Choosing quercetin as the main model for docking analyses makes sense because it represents the basic structure of flavonols found in most compounds in the mistletoe. Moreover, most of the identified phenolic compounds share a common structural motif derived from caffeic acid (**Supplementary Table 1**).

### 3.2 Target enzymes

To further explore the potential of *V. album* L. leaves extract as a source of anti-*H. pylori* agents, we evaluated the binding affinity of its most abundant phenolic and flavonoid constituents—caffeic acid and quercetin—against essential *H. pylori* enzymes using molecular docking simulations.

One of our primary targets was peptide deformylase (HpPDF), a key metalloenzyme involved in bacterial protein maturation. HpPDF catalyzes the removal of the N-terminal formyl groufrom nascent polypeptides, a critical stein protein biosynthesis unique to bacteria (Griffith et al., 1976; Torzewska et al., 2003). Importantly, this enzyme is absent in humans, making it an attractive and selective target for antimicrobial drug development. Previous studies have demonstrated the viability of HpPDF as a drug target, with several promising inhibitors already identified (Han et al., 2004; Cai et al., 2006; Saravanakumar et al., 2019).

**Figure 1:**
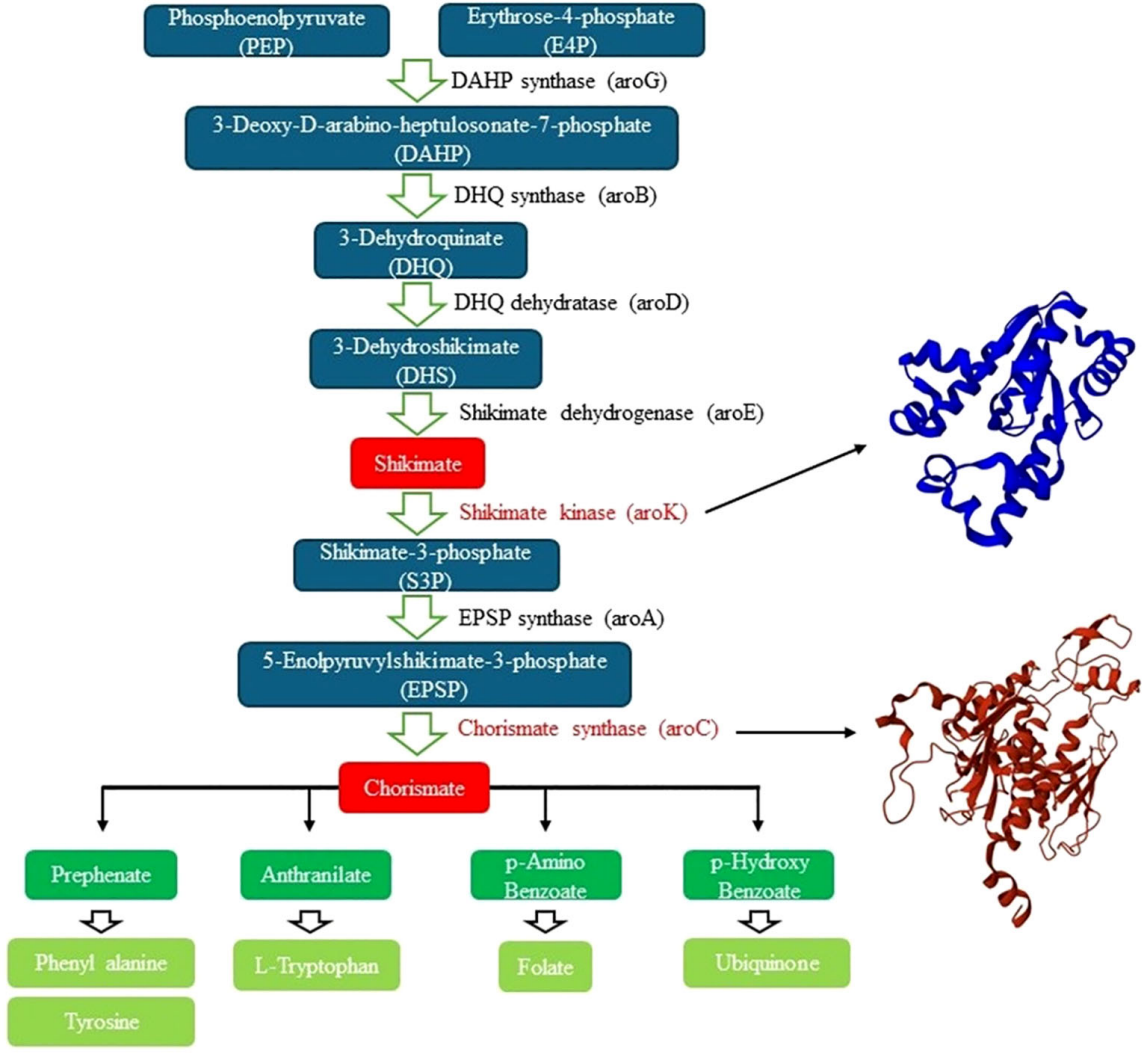
Schematic representation of the shikimate pathway in *H. pylori* and the targeted enzymes involved in aromatic amino acid biosynthesis.

We also focused on disrupting the shikimate pathway, a vital biosynthetic route in *H. pylori* that is absent in humans. This seven-steenzymatic cascade (**Figure 1**) is essential for the synthesis of aromatic amino acids such as phenylalanine, tyrosine, and tryptophan (Cheng et al., 2005; Singh et al., 2022; Noori Goodarzi et al., 2024). Given its indispensable role in bacterial metabolism and its absence in mammals, the shikimate pathway represents a rich source of selective drug targets (Cheng et al., 2005; Noori Goodarzi et al., 2024). Within this pathway, we selected two key enzymes for docking studies: shikimate kinase and chorismate synthase. Shikimate kinase catalyzes the ATP-dependent phosphorylation of shikimate to shikimate-3-phosphate, a critical stetoward the production of chorismate (Cheng et al., 2005; Han et al., 2007). Chorismate synthase, the final enzyme in this pathway, converts 5-enolpyruvylshikimate-3-phosphate into chorismate, the common precursor for a range of aromatic metabolites (Ahn et al., 2004; Celli et al., 2009; Ibrahim et al., 2020; Al Khzem et al., 2025).

The pathway begins with phosphoenolpyruvate (PEP) and erythrose-4-phosphate (E4P) and proceeds through seven enzymatic steps leading to the production of chorismate, a key precursor for essential metabolites. Two critical enzymes were selected for molecular docking studies: shikimate kinase (aroK) and chorismate synthase (aroC), whose 3D structures are shown on the right panel: 3N2E (Cheng et al., 2012) for shikimate kinase and 1UMF (Ahn et al., 2004) for chorismate synthase. The shikimate pathway is absent in humans but essential for microbial viability, making its enzymes attractive targets for antimicrobial drug development.

**Figure 2:**
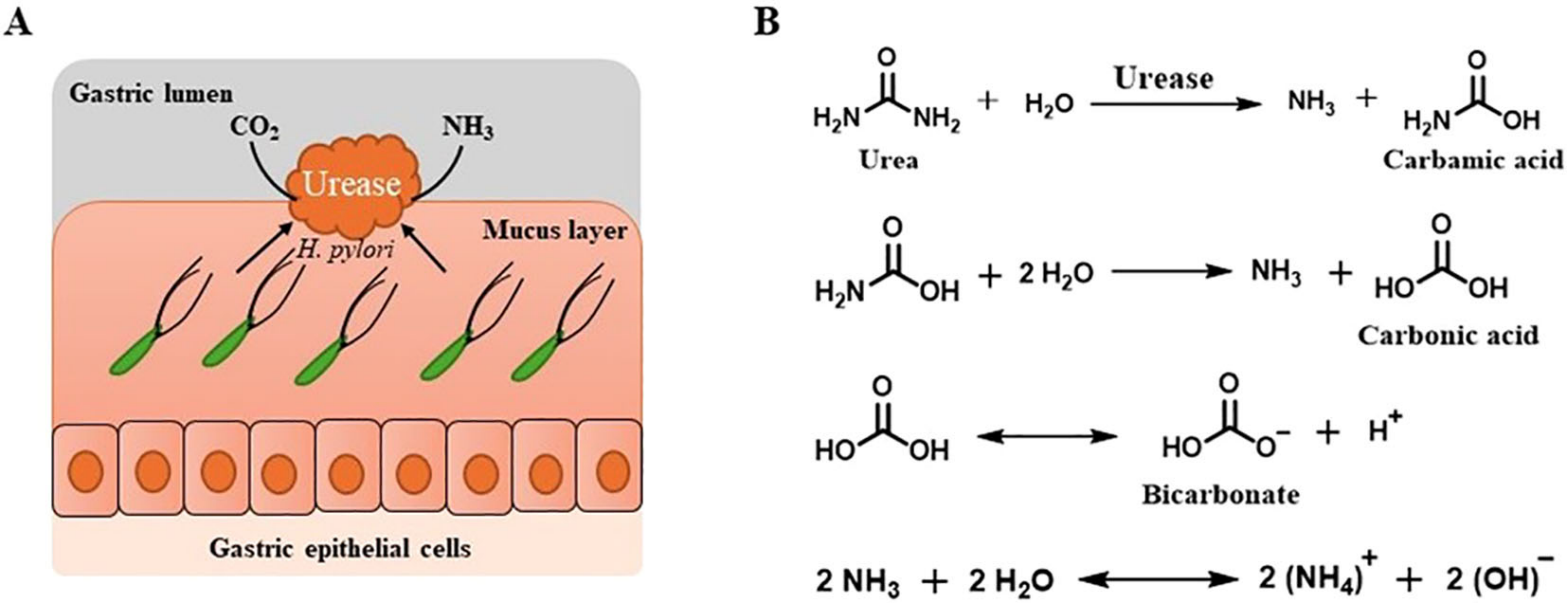
The role of Urease enzyme in the survival and colonization of *H. pylori* in the gastric mucosa. **(A)** Role of urease in the pathogenesis of *H. pylori*. **(B)** Hydrolysis of urea by urease to yield ammonia and carbonic acid.

*H. pylori* produce urease, a nickel-dependent metalloenzyme that plays a pivotal role in the bacterium’s survival and pathogenicity. This enzyme enables *H. pylori* to withstand the harsh acidic conditions of the gastric environment by catalyzing the hydrolysis of urea into ammonia and carbamate (**Figure 2**). The resulting ammonia neutralizes gastric acid, thereby creating a more hospitable niche for colonization and persistent infection (Celli et al., 2009; Cui et al., 2013; Olivera-Severo et al., 2017; Cunha et al., 2021).

The binding affinity and binding mechanisms for the tested compounds, caffeic acid, and quercetin on four proteins associated with *H. pylori* were investigated using a molecular docking approach. The tested ligands showed variable binding affinities to the target proteins under investigation, as indicated by the docking score (**Table 4**). The binding of the tested compounds showed different binding forces including both hydrophobic interactions and hydrogen bonding. In addition, the metal containing ligands showed additional charge attraction and metal-acceptor bonding.

**Table 4:** Docking affinities (Kcal/mol) for studied compounds into proteins of *H. pylori*.

Protein	Urease (1E9Y)	Chorismate synthase (1UMF)	Peptide deformylase (2EW5)	Shikimate kinase (3N2E)
Quercetin	-6.3	-8.5	-7.8	-8.6
Naringenin	-5.6	-7.7	-7.5	-8.2
Apigenin	-5.8	-8.1	-7.7	-8.6
Caffeic acid	-6.4	-6.1	-5.9	-6.3
Gallic acid	-5.7	-6.9	-5.6	-6.1
Silver Azelate	-6.4	-5.9	-5.7	-6.4
Selenium Azelate (1:1)	-5.0	-6.2	-7.3	-6.5

### 3.3 Molecular docking analysis of caffeic acid and quercetin with *H. pylori* peptide deformylase

**Figure 3:**
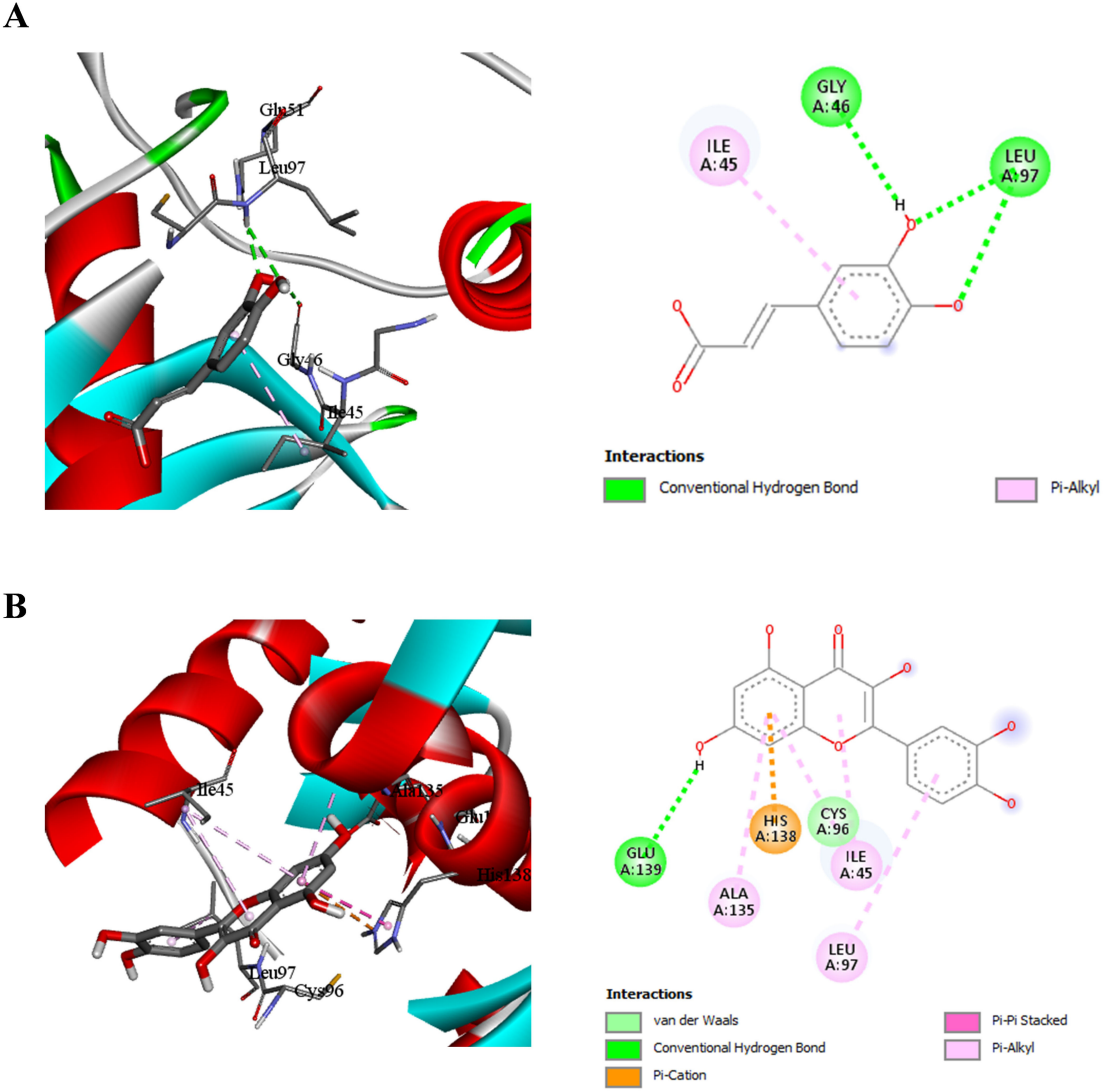
Molecular docking interactions of caffeic acid **(A)** and quercetin **(B)** with *H . pylori* peptide deformylase (code: 2EW5). Each panel shows the 3D binding conformation (left) and 2D interaction ma(right) highlighting hydrogen bonds, hydrophobic, π interactions, and other relevant molecular contacts.

**Figure 3** presents the docking results for caffeic acid and quercetin with *H. pylori* peptide deformylase. Caffeic acid (**Figure 3A**) forms conventional hydrogen bonds with Gly46, and Leu97, stabilizing the ligand within the catalytic cleft. A π-alkyl interaction with Ile45 further supports the hydrophobic engagement of the ligand.

Quercetin (**Figure 3B**) shows a richer interaction profile including hydrogen bonding with Glu139, π-cation interactions with His138, π-stacking with Ala135, and π-alkyl and van der Waals interactions with Ile45, Leu97, and Cys96. This multifaceted interaction network indicates a strong inhibitory potential of quercetin through effective binding and possible distortion of the catalytic machinery.

Confirming our findings other studies found that caffeic acid esters like Caffeic acid phenethyl ester (CAPE) from other natural sources like propolis efficiently inhibits *H. pylori* peptide deformylase activity (Cui et al., 2013).

### 3.4 Molecular docking analysis of caffeic acid and quercetin with *H. pylori* shikimate kinase

**Figure 4:**
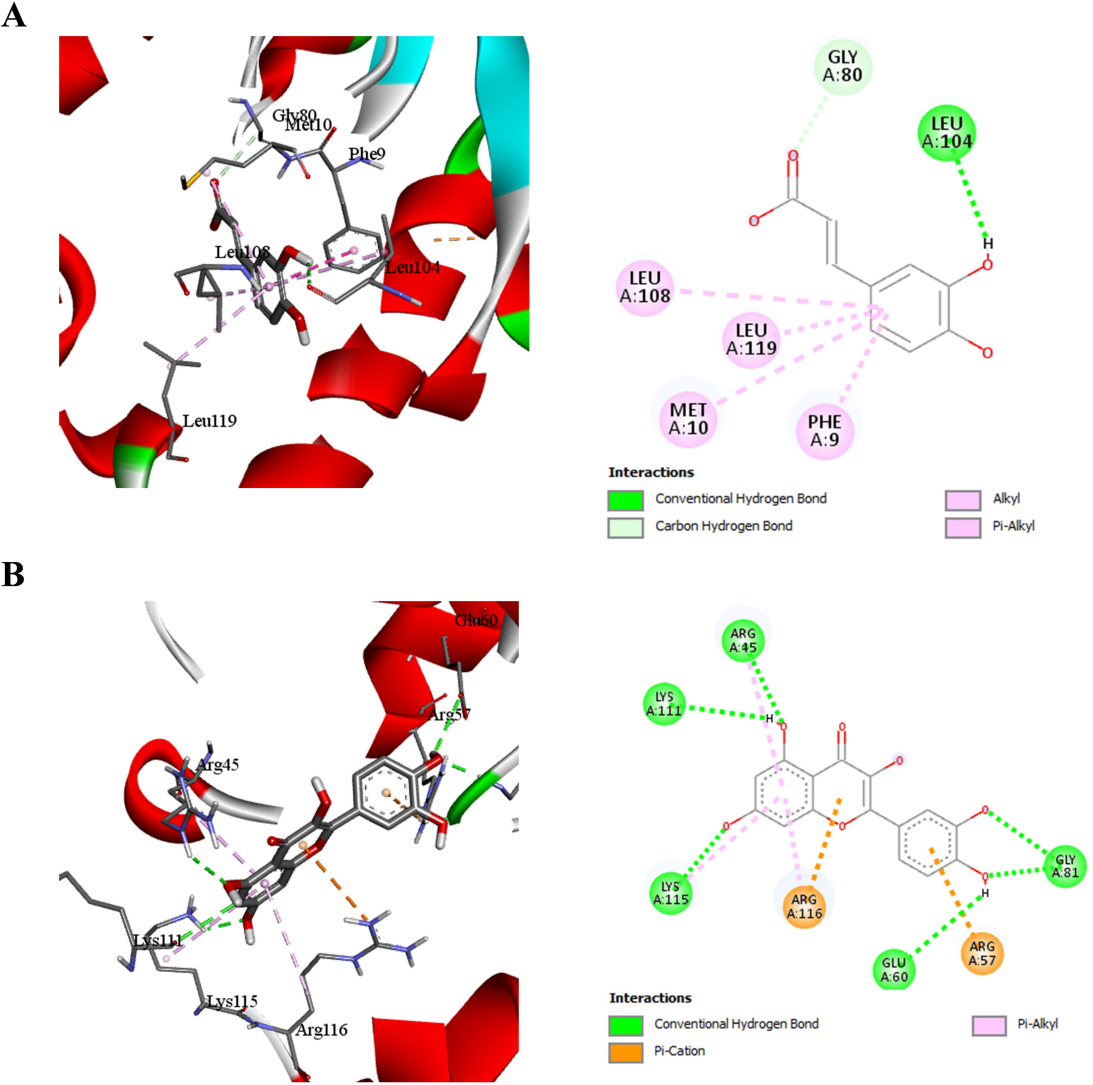
Molecular docking interactions of caffeic acid **(A)** and quercetin **(B)** with *H. pylori* Shikimate Kinase (code: 3N2E).

Docking studies revealed distinct binding profiles of caffeic acid and quercetin with *H. pylori* shikimate kinase (**Figure 4**).

Caffeic acid (**Figure 4A**) formed several non-covalent interactions, notably conventional hydrogen bonds with Gly80, and Leu104, which are crucial residues lining the binding cavity. Additional hydrophobic interactions were observed with Leu108, Leu119, Met10, and Phe9, enhancing ligand stabilization *via* alkyl and π-alkyl interactions. These interactions suggest that caffeic acid has the capacity to occupy and stabilize within the substrate-binding pocket.

Quercetin (**Figure 4B**), however, exhibited a more extensive interaction profile. It engaged in hydrogen bonding with Lys115, Lys111, Gly81, Glu60, and Arg45, and established π-cation interactions with Arg116 and Arg57, which are key residues adjacent to the ATP-binding motif. The presence of both polar and electrostatic interactions, in combination with hydrophobic contacts, indicates that quercetin can establish strong affinity within the active site, suggesting its potential as a more robust inhibitor than caffeic acid.

### 3.5 Molecular docking analysis of caffeic acid and quercetin with *H. pylori* chorismate synthase

**Figure 5:**
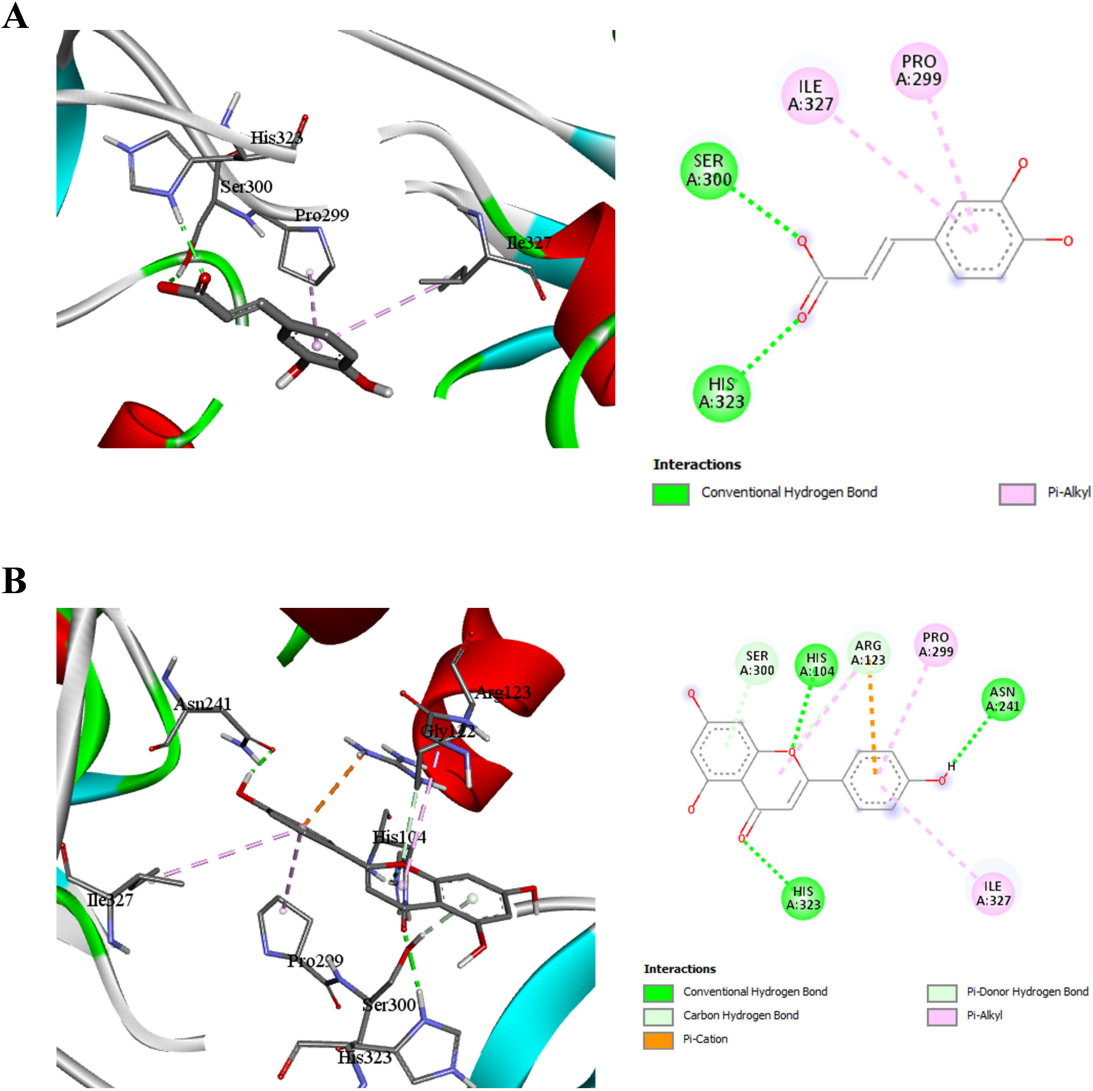
Molecular docking interactions of caffeic acid **(A)** and quercetin **(B)** with *H. pylori* Chorismate Synthase (code: 1UMF).

Molecular docking results of caffeic acid and quercetin with *H. pylori* chorismate synthase are shown in **Figure 5**.

Caffeic acid (**Figure 5A**) interacts *via* conventional hydrogen bonds with His323, Ser300, and His274, and forms π-alkyl interactions with Ile327, and Pro299. These interactions are predominantly within the hydrophobic core of the active site, suggesting moderate stabilization.

Quercetin (**Figure 5B**) showed enhanced binding *via* multiple interaction types, including hydrogen bonds with Asn241, His323, and His104, and π-cation and π-donor hydrogen bonds with Arg123, Ser300, His104, and Ile327. The wider range of interactions suggests quercetin exhibits greater binding specificity and potential inhibitory strength against chorismate synthase.

### 3.6 Molecular docking analysis of caffeic acid and quercetin with *H. pylori* urease

**Figure 6:**
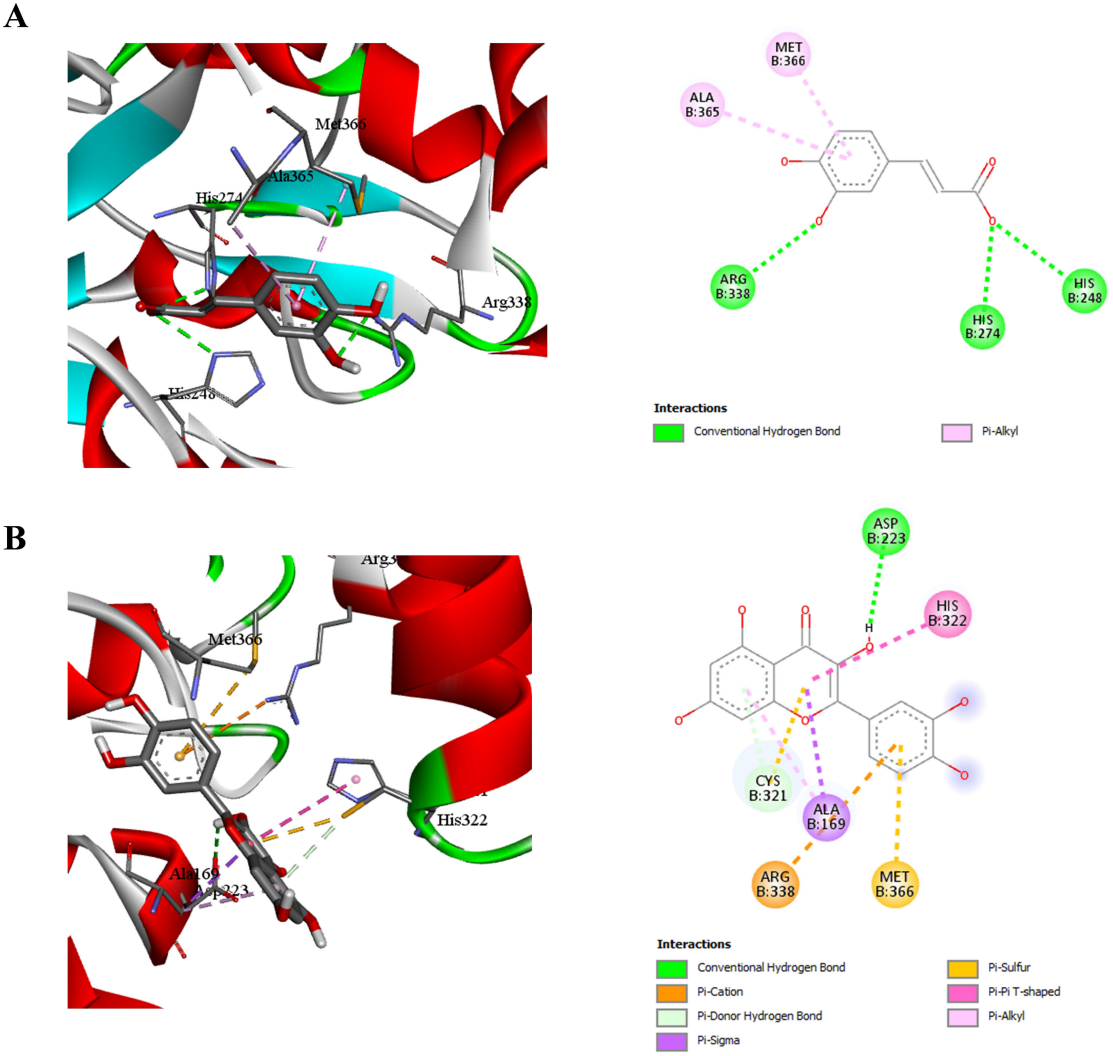
Molecular docking interactions of caffeic acid **(A)** and quercetin **(B)** with *H. pylori* Urease (code: 1E9Y).

**Figure 6** illustrates the docking interactions of caffeic acid and quercetin with *H. pylori* urease.

Caffeic acid (**Figure 6A**) exhibited hydrogen bonding with Arg338, His274, and His248, and π-alkyl interactions with Met366, and Ala365. These interactions indicate strong affinity for the nickel-containing catalytic core. Quercetin (**Figure 6B**) bound deeply within the urease active site, forming conventional hydrogen bonds with Cys321, and Asp223, and π-cation/π-stacked interactions with Arg338, Met366, His322, and Ala169. This complex interaction pattern supports quercetin’s strong inhibitory potential by occupying and potentially distorting the urease active site configuration.

To date, numerous urease inhibitors have been screened and designed, such as hydroxamic acids, quinones, polyphenolics, and other heterocyclic compounds (Kafarski and Talma, 2018; Szczerbiec et al., 2024). Surprisingly, Hydrogen bond formations were considered as most important aspect for perfect fitting of ligand within the enzyme and this seems the case of caffeic acids and quercetin of *V. album* extract.

### 3.7 *In vitro* activity against urease of *P. mirabilis*

Urease is central to *H. pylori* metabolism and virulence. Also, urease has been demonstrated as a potent virulence factor for some species, including *P. mirabilis* (Jones et al., 1990), and *Staphylococcus saprophyticus (*Gatermann and Marre, 1989). In *H. pylori*, urease consists of two subunits: *UreA* (alpha) and *UreB* (beta) (Clayton et al., 1990; Labigne et al., 1991) whereas in *P. mirabilis*, it comprises three subunits: *UreA* (gamma), *UreB* (beta), and *UreC* (alpha) (Jones and Mobley, 1989; Fitzgerald et al., 2024).

**Figure 7:**
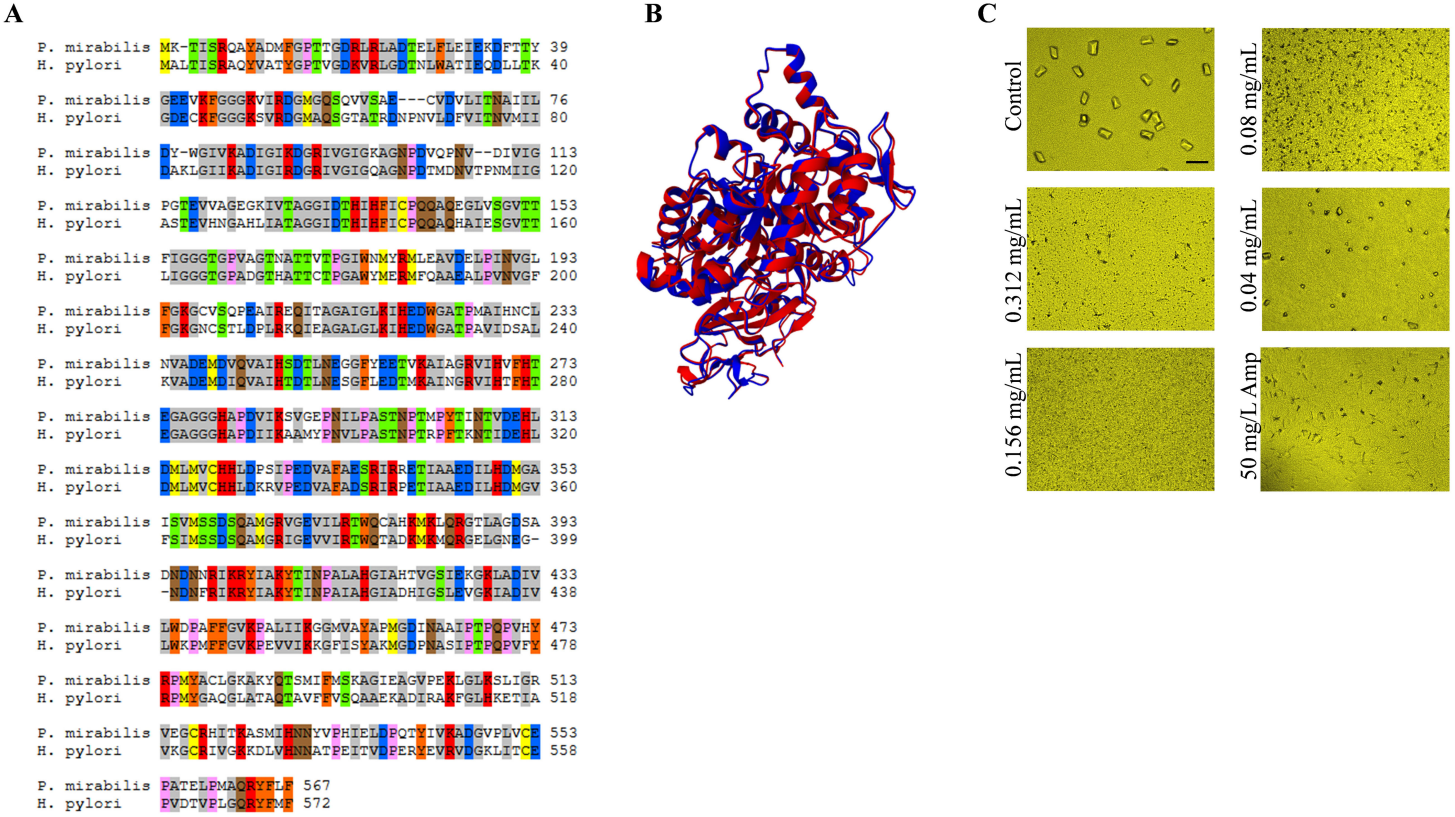
Urease inhibition by *V. album* extract in *P. mirabilis*. **(A)** Pairwise sequence alignment of *UreB* (GAA7509432.1) from *H . pylori* and *UreC* (CAR47185.1) from *P. mirabilis* strain HI4320 (Pearson et al., 2008). Conserved residues are highlighted in color, suggesting partial sequence identity and conservation of functional motifs. Sequences were aligned and colored using the sequence manipulation suite (Stothard, 2000) **(B)** Structural superimposition of the predicted 3D structures by AlphaFold v2.3.2 (AF2) (Evans et al., 2021) of *H . pylori UreB* (blue) and *P. mirabilis UreC* (red), demonstrating overall structural similarity and conserved folding patterns. The 2 proteins were superimposed and visualized using Mol* Viewer (Sehnal et al., 2021). **(C)** Dose dependent effect of *V. album* extract on urease-mediated crystal formation by *P. mirabilis*. Microscopic images show reduced crystal formation at increasing extract concentrations compared with untreated control. Ampicillin (50 mg/L) served as reference control to inhibit bacterial growth. Scale bar = 50 µm.

The *UreB* of *H. pylori* share more than 40% identity with *UreC* of *P. mirabilis* (**Figure 7A**), moreover the 2 enzymes showed perfect superimposition of their 3D structures (**Figure 7B**).

The conserved nature of the catalytic domains in these urease subunits implies potential for cross-species inhibition strategies. Inhibitors designed to target these conserved regions may exhibit efficacy against both *H. pylori* and *P. mirabilis*, offering a broader spectrum of antibacterial activity.

The ethanol extract of *V. album* exhibited bactericidal activity against *P. mirabilis* with an MIC of 1.56 ± 0.24 mg/mL. Additionally, it demonstrated strong anti-urease effects at concentrations as low as 0.0125 mg/mL, highlighting its potent inhibitory action on urease. In addition to direct urease inhibition, *V. album* extract was evaluated for its effect on urease-mediated crystal formation in artificial urine. Struvite (magnesium ammonium phosphate hexahydrate; MgNH_4_PO_4_·6H_2_O) is the predominant crystalline component, and its formation occurs at alkaline conditions (pH ≥ 7.2), driven by increase in the concentration of CO3^2−^ and NH4^+^ ions through urease activity (Prywer and Torzewska, 2012; Prywer et al., 2012; Li et al., 2015; Prywer et al., 2018), according to the following equation.


$$Mg^{2+}+\;{NH}_{4}^{+}+\;PO_{3}^{-4}+\;6H_{2}O\to 2MgNH_{4}PO_{4}\cdot 6H_{2}O\;(↓);\;pH\geq 7$$

Natural compounds such as curcumin and vanillic acid have demonstrated inhibitory effects on struvite crystallization *in vitro* (Pearson et al., 2008; Prywer and Torzewska, 2012). We grew urease producing *P. mirabilis* in artificial urine with escalating sub MIC doses of *V. album* extract (0.312 to 0.04 mg/mL), as a control to inhibit bacterial growth we incorporated ampicillin (50 mg/L), aside from positive control of crystal formation which is untreated neither with the extract nor the antibiotic. Qualitative assessment by microscopic examination demonstrated that untreated samples exhibited dense crystal aggregation, whereas treatment with increasing concentrations of *V. album* markedly reduced crystal deposition in a concentration-dependent manner (**Figure 7C**). At 1/5^th^ MIC (0.312 mg/mL), only sparse or no crystals were detected, indicating significant suppression of urease-driven precipitation.

Struvite stones can grow rapidly, often occupying the entire renal pelvis and calyces, potentially causing urinary tract obstruction and serious complications, including loss of kidney function (Singh et al., 1973; Wojewski and Zajaczkowski, 1973). In the same context, treatment of infectious urolithiasis is long-term and complex, involving antibiotics to eradicate the causative pathogen, stone removal *via* shock wave lithotripsy (SWL) or percutaneous nephrolithotomy (PCNL), and measures to prevent recurrence. Antibiotic therapy is often challenging, as drugs may not penetrate the stone matrix, allowing bacteria to survive and contribute to recurrent infections and *de novo* stone formation (Bichler et al., 2002; Barthel and Markwardt, 1975; Marien and Miller, 2015). Our findings suggest that *V. album* could serve as an alternative strategy to prevent urolithiasis by inhibiting urease activity.

### 3.8 Extended anti-virulence potential of *V. album* extract

Finally, we decided to test the anti-virulence activities of *V. album* against a subset of pathogens like *E. coli*, *S. aureus* and *P. aeruginosa*.

Bacterial biofilms are structured communities of microorganisms attached to surfaces and encased in a self-produced extracellular polymeric substance (EPS) matrix. Composed of polysaccharides, proteins, nucleic acids, and lipids, this matrix provides structural support, facilitates communication and nutrient exchange, and protects bacteria from environmental stressors such as antibiotics, desiccation, and host immune responses (Singh et al., 2021; El-Telbany et al., 2022). It worth mentioning that biofilm formation is associated with increased resistance to eradication of *H. pylori*, contributing to the persistence of infection and the progression of peptic ulcers (Elshenawi et al., 2023).

**Figure 8:**
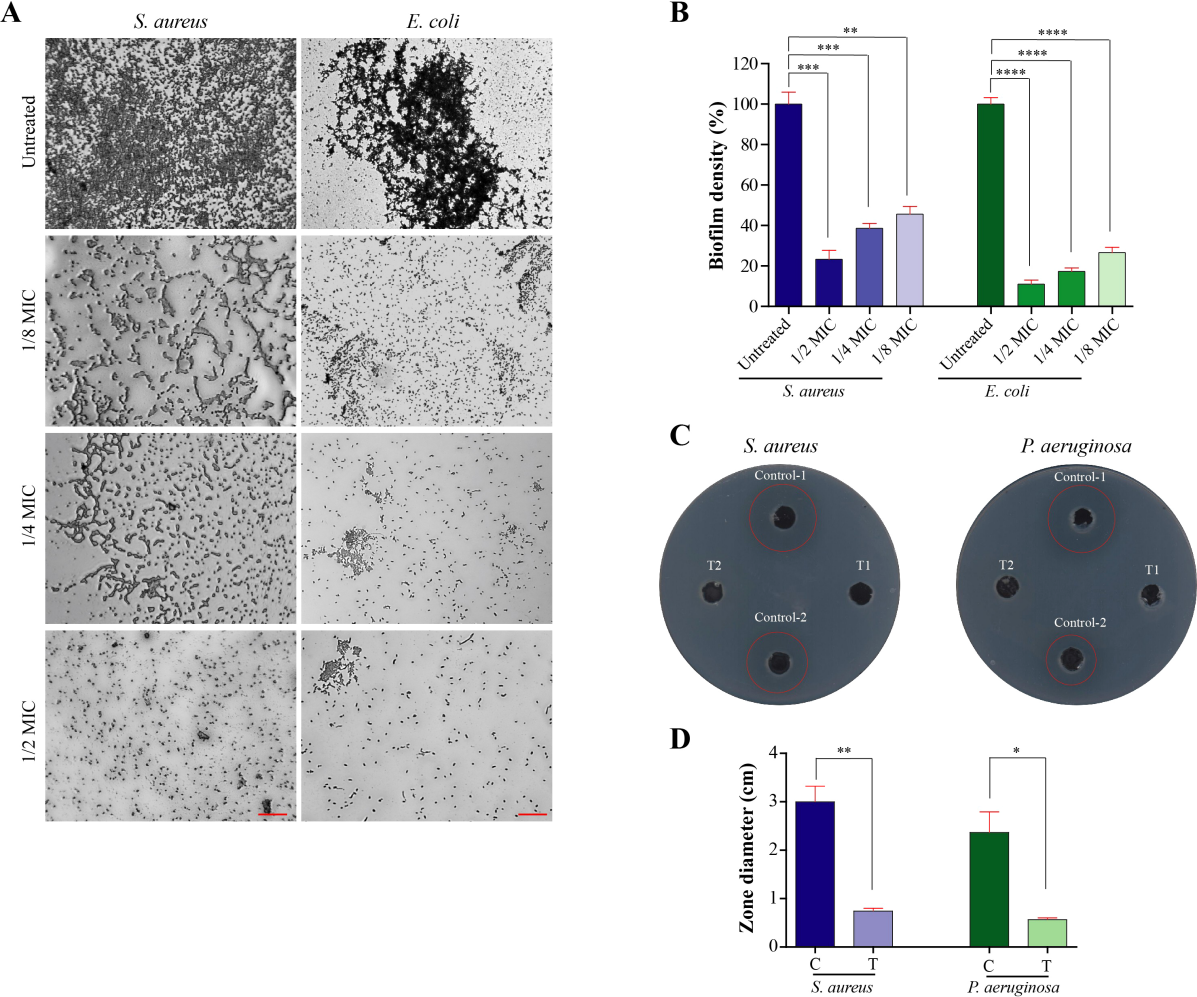
Extended anti-virulence effects of *V. album* extract against biofilm formation and protease secretion in pathogenic bacteria. **(A)** Microscopic visualization of biofilm inhibition in *S. aureus* and *E coli* following treatment with sub-MIC concentrations of *V. album* extract (1/8, 1/4, and 1/2 MIC) compared with untreated controls. Scale bar = 50 µm. **(B)** Quantification of biofilm biomass showing significant, concentration-dependent reduction in biofilm density in *S. aureus* and *E coli*. **(C)** Protease inhibition assay in *S. aureus* and *P. aeruginosa*, showing clear inhibition zones around wells loaded with supernatants from *V. album* treated (T1, T2) or untreated control cultures (Control-1, Control-2). **(D)** Measurement of inhibition zone diameters demonstrating significant protease inhibition in *S. aureus* and *P. aeruginosa* following *V. album* treatment (C, control; T, treated). Data are mean ± SEM (n = 3); *< 0.05, **< 0.01, ***< 0.001, ****< 0.0001.

We assessed the antibiofilm efficacy of *V. album* extract at sub-MIC doses by comparing biofilm density in treated versus untreated cultures. Microscopic imaging revealed that untreated *S. aureus* (MIC = 2.68± 0.44 mg/mL) and *E. coli* (MIC = 3.12± 0.36 mg/mL) formed dense and structured biofilms, whereas treatment with sub-MIC concentrations of *V. album* extract led to a concentration-dependent disruption of biofilm architecture (**Figure 8A**). At ½ MIC (1.34 mg/mL for *S. aureus* and 1.56 mg/mL for *E. coli*), only sparse and scattered cells were observed, indicating strong inhibition of biofilm matrix development. Quantitative analysis confirmed these findings, with biofilm density significantly reduced in both *S. aureus* (80% loss of biofilm density at ½ MIC dose) and *E. coli* (90% loss of biofilm density at ½ MIC dose) in a dose-dependent manner (**Figure 8B**).

The observed antibiofilm activity of the *V. album* extract aligns with previous studies demonstrating that plant-derived polyphenols can disrupt biofilm formation in Gram-positive and negative bacteria (Kurt et al., 2023; Alum et al., 2025; Zhang et al., 2025). For example, quercetin and related flavonoids have been reported to inhibit biofilm development in *S. aureus and E. coli* by interfering with quorum-sensing pathways and reducing extracellular polymeric substance (EPS) production (Lee et al., 2013; Júnior et al., 2018). Similarly, caffeic acid and other phenolic acids have shown significant antibiofilm effects against *S. aureus, E. coli, aeruginosa and Klebsiella pneumonia* (Bhattacharya and Mandal, 2025). These findings are consistent with our results, where the extract and its major constituents reduced biofilm biomass and impaired bacterial adherence. Collectively, this suggests that the antibiofilm activity observed in our study is in line with the broader antimicrobial profile of these phytochemicals and highlights their potential applicability against persistent *H. pylori* biofilms.

Proteases, another key bacterial virulence factor, contribute to infection by degrading host tissues and facilitating immune evasion (Cul and Wright, 2017; Qandeel et al., 2025). To examine the effect of *V. album* extract on protease activity, cultures of *P. aeruginosa* (MIC = 3.78± 0.54 mg/mL) and *S. aureus* were treated with sub MIC doses of the extract (½, ¼, and ⅛^th^) and analyzed using the skim milk agar method. Supernatants from untreated cultures displayed strong protease activity, evidenced by clear zones surrounding the wells (**Figures 8C, D**). In contrast, treated cultures showed complete absence of inhibition zones, indicating full suppression of protease activity.

Although direct reports on *V. album* protease inhibition are limited, previous studies have demonstrated that its phenolic constituents including flavonoids such as quercetin and phenolic acids such as caffeic and gallic acids possess significant inhibitory effects on microbial and bacterial proteases (Joshi et al., 2015; Zhang et al., 2025). For xample, quercetin has been reported to inhibit bacterial metalloproteases and serine proteases (Nguyen and Bhattacharya, 2022), thereby reducing virulence and tissue invasion. Similarly, caffeic acid and related hydroxycinnamic acids display dose-dependent inhibition of bacterial protease activity (Daglia, 2012; Miklasińska-Majdanik et al., 2018).

Together, these results expand the therapeutic relevance of *V. album*, showing that its effects extend beyond growth inhibition to direct suppression of virulence traits. Such multi-targeted anti virulence activity makes *V. album* a promising candidate for adjunctive therapies aimed at mitigating pathogenicity while potentially reducing selective pressure for antibiotic resistance.

## 4 Conclusions

This study provides a comprehensive *in silico* and *in vitro* evaluation of *V. album* (mistletoe) extract as a potential antimicrobial agent targeting key enzymatic systems in *H. pylori*, *P. mirabilis*, and other pathogens. Molecular docking revealed strong binding affinities of caffeic acid and quercetin—major phenolic and flavonoid components of the extract—against essential *H. pylori* enzymes, including peptide deformylase and key enzymes of the shikimate pathway (shikimate kinase and chorismate synthase). These targets are critical for bacterial survival and are absent in humans, enhancing the therapeutic selectivity of the proposed inhibitors. *In vitro* assays validated the inhibitory action of *V. album* against urease from *P. mirabilis*. Importantly, urease inhibition translated into a significant reduction in crystal formation, underscoring the clinical relevance of this effect in urinary tract infections. Moreover, *V. album* exhibited extended anti virulence activity across pathogens, disrupting biofilm formation in *S. aureus* and *E. coli*, and suppressing protease activity in *S. aureus* and *P. aeruginosa*. These combined results establish *V. album* phenolics as dual-action inhibitors that both target essential bacterial enzymes and attenuate key virulence traits, offering a promising natural therapeutic approach against multidrug-resistant bacteria. Future pharmacological studies and *in vivo* validations are warranted to advance the development of *V. album*-derived compounds as plant-based antimicrobials.

## Data Availability

The original contributions presented in the study are included in the article/**Supplementary Material**. Further inquiries can be directed to the corresponding authors.
