# Supplementary material for: Targeting Helicobacter pylori enzymes using Viscum album L. extract: in silico molecular docking and in vitro study
Source: Front Cell Infect Microbiol. 2026 Jan 12;15:1690969. doi: 10.3389/fcimb.2025.1690969 (PMC12832787; doi:10.3389/fcimb.2025.1690969)
Supplement: Supplementary file 1 [file Supplementaryfile1.docx]

Supplementary material

Calibration curves

**Chromatograms**

** 280 nm**

**340 nm**
