# Supplementary material for: Targeting Helicobacter pylori enzymes using Viscum album L. extract: in silico molecular docking and in vitro study
Source: Front Cell Infect Microbiol. 2026 Jan 12;15:1690969. doi: 10.3389/fcimb.2025.1690969 (PMC12832787; doi:10.3389/fcimb.2025.1690969)
Supplement: Supplementary file 2 [file Supplementaryfile2.docx]

**Supplementary material**

**Chemical structures**
The 2D chemical structures of the main compounds were retrieved from publicly available databases, including PubChem (<https://pubchem.ncbi.nlm.nih.gov>) and ChemSpider (<https://www.chemspider.com>)

**Table 1s. Chemical structure of the main phenolic compounds identified in mistletoe leaves extract**

| 1.  Dihydroxybenzoic acid | 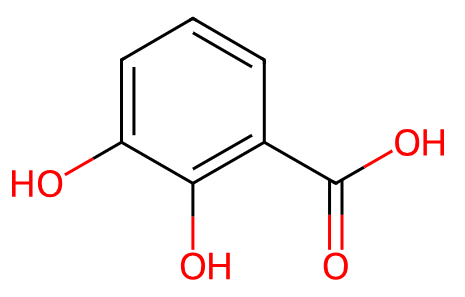 |
| --- | --- |
| 2.  3-Caffeoylquinic acid | 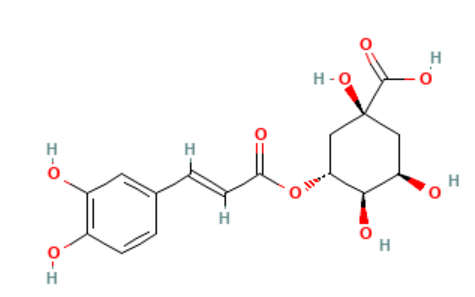 |
| 3.  4-Caffeoylquinic acid | 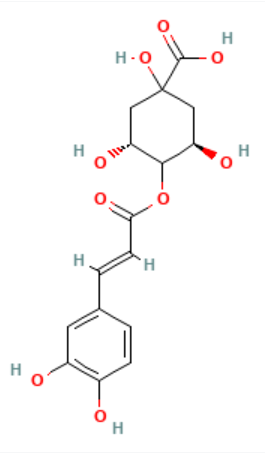 |
| 4.  5-Caffeoylquinic | 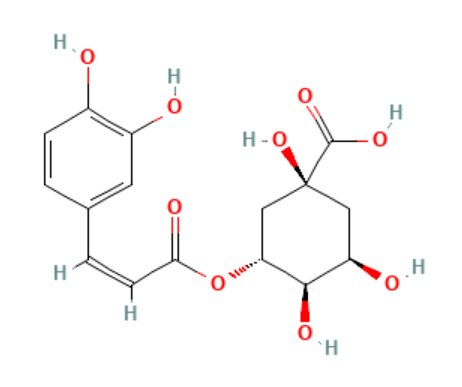 |
| 5. Sinapic acid glucoside | 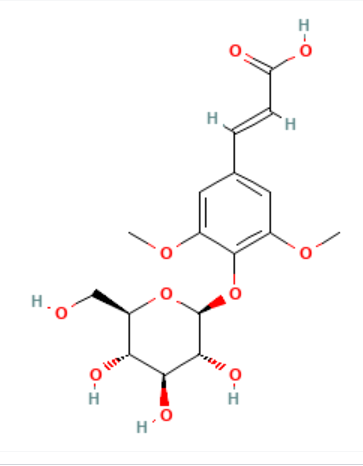 |
| 6. Dicaffeoyl -tartaric acid | 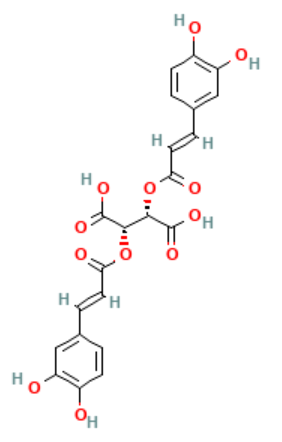 |
| 7. 3-Sinapoylquinic acid | 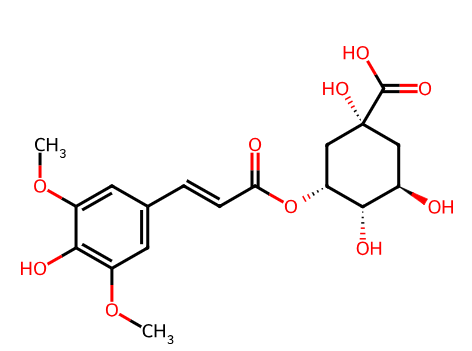 |
| 8.  5-Sinapoylquinic acid | 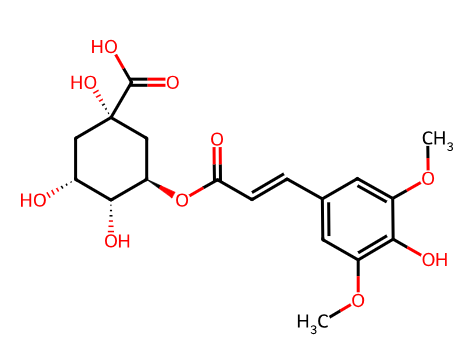 |
| 9. Quercetin-rutinoside  (Rutin) | 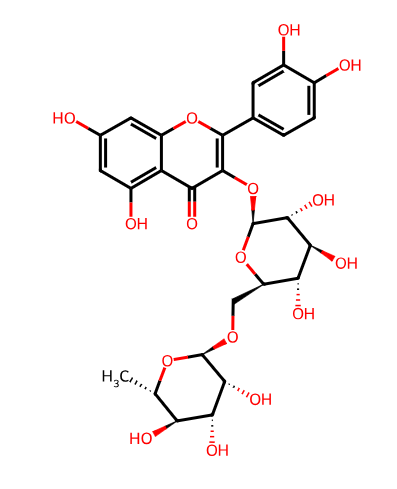 |
| 10.  Quercetin-glucoside | 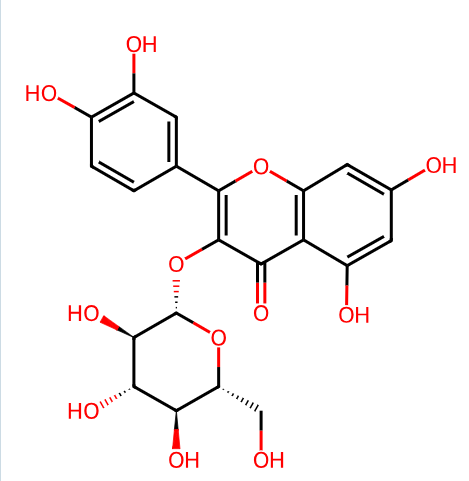 |
| 11. Sinapic acid | 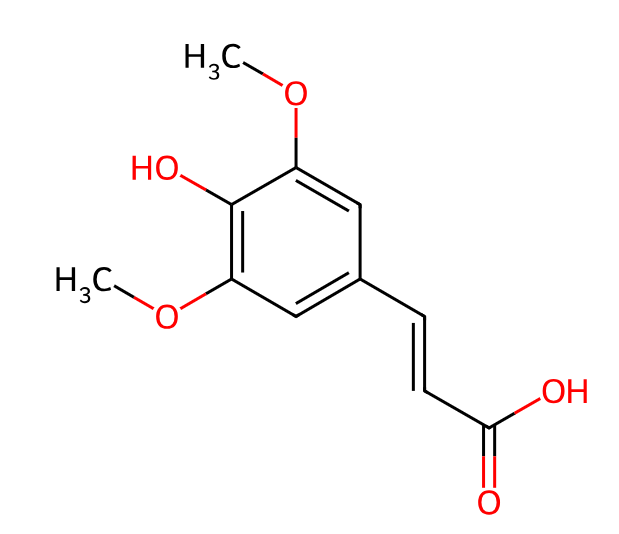 |
| 12.  Quercetin-O-[hydroxymethylglutaryl] hexoside |  |
| 13.  Isorhamnetin-glucoside | 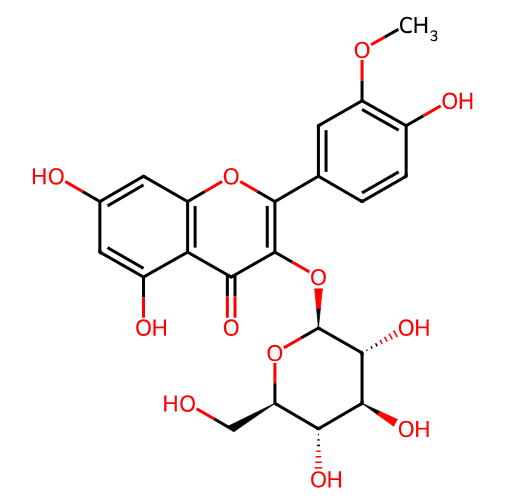 |
| 14 |  |
| 15 Isorhamnetin-glucuronide | 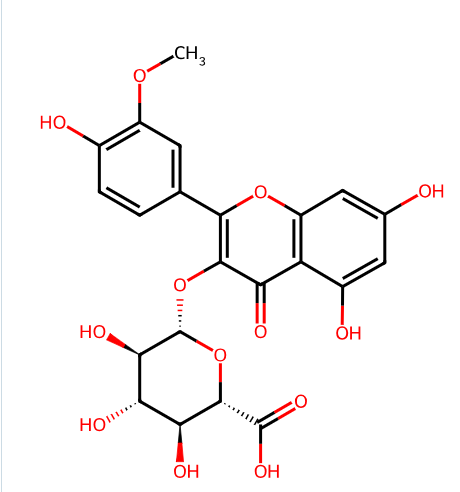 |
| 16 |  |
| 17  Isorhamnetin-glucosyl-rhamnoside | 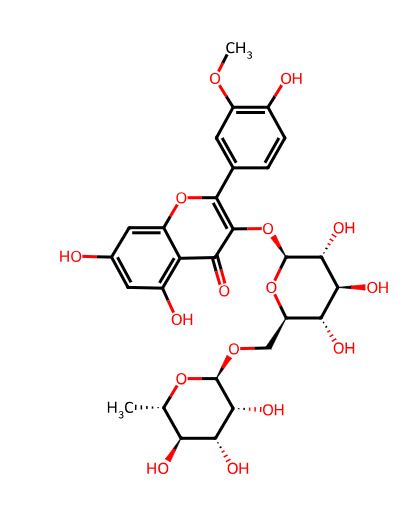 |
| 18 |  |
| 19  Rhamnazin-rutinoside | 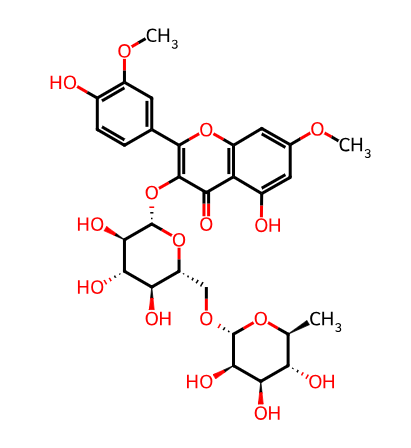 |
| 20 Quercetin | 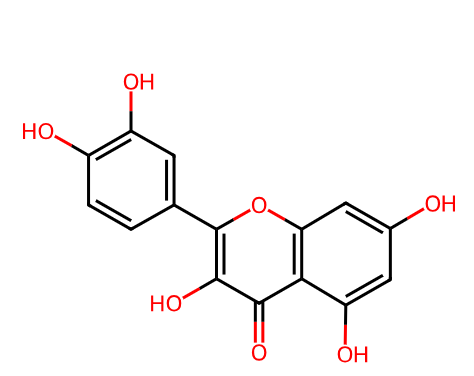 |
| 21 Isorhamnetin | 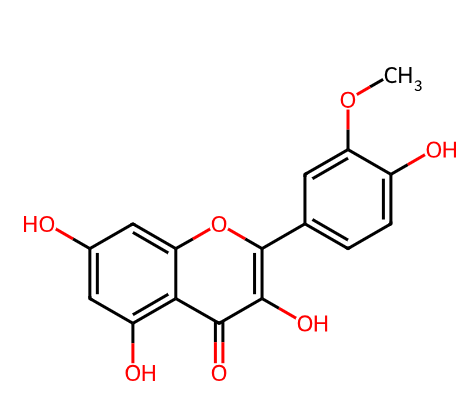 |

Compounds 2, 3, 4 and 6 (3-caffeoylquinic acid, 4-caffeoylquinic acid, 5-caffeoylquinic acid and dicaffeoyl-tartaric acid) share a common structural motif derived from caffeic acid.
This motif consists of a *catechol ring* (3,4-dihydroxyphenyl) linked to a *conjugated propenoic chain* (–CH=CH–COOH), representing the characteristic 3,4-dihydroxycinnamic backbone of caffeic acid.
In all identified esters, this caffeic-acid–derived fragment is preserved and esterified with quinic or tartaric acid.

The presence of this caffeic acid moiety is functionally relevant, as the catechol group provides two ortho-hydroxyls capable of rapid electron donation, metal chelation, and resonance stabilization of phenoxyl radicals, thereby contributing to the antioxidant properties of these compounds.
